# SARS-CoV-2 infection after vaccination in Italian health care workers: a case report

**DOI:** 10.1007/s40009-022-01106-w

**Published:** 2022-03-06

**Authors:** Concetta Cafiero, Raffaele Palmirotta, Alessandra Micera, Maria Pia Ottaiano, Agnese Re, Florinda Pedata, Domenico Costagliola, Domenico Ponticelli, Salvatore Pisconti, Beniamino Schiavone, Giulio Benincasa

**Affiliations:** 1 Medical Oncology, SG Moscati Hospital, Taranto, Italy; 2 Interdisciplinary Department of Medicine, School of Medicine, University of Bari ‘Aldo Moro’, Piazza G. Cesare, 11, 70124 Bari, Italy; 3 Research Laboratories in Ophthalmology, IRCCS - Fondazione Bietti, via Santo Stefano Rotondo 6, 00184 Rome, Italy; 4 Department of Clinical Pathology and Molecular Biology, Pineta Grande Hospital Castel Volturno (CE), Castel Volturno (CE), Italy; 5 Catholic University of Sacred Heart, Rome, Italy; 6 Pineta Grande Hospital, Castel Volturno (CE), Italy

**Keywords:** Post-vaccinal infection, COVID-19, Healthcare workers, Genetic variants

## Abstract

Following the approval of COVID-19 vaccination program by EMA and national authorities, an immunization campaign started in Italy with BNT162b2mRNA vaccine, initially focused on healthcare workers. The active immunization was monitored by systemic antibody titration and continuous surveillance was guaranteed by antigenic/molecular tests on swabs. Cases of infection have been recently observed in vaccinated healthcare workers. Herein we describe an outbreak of infection occurring in five physicians out of 656 healthcare workers belonging to a private hospital, referring mild symptoms of COVID-19. Healthcare workers underwent complete vaccination and screening for antibody titration. Five out of 656 healthcare workers were tested positive for SARS-CoV-2 in nasopharyngeal swabs and referred mild COVID-19 symptoms. Molecular analyses were carried out to identify possible variants of Spike protein. Their genotyping performed on RNA extracts highlighted the presence of del69/70, N501Y, A570D, and 1841A > G (D614G) sequence variants, all indicative of VOC 202012/01-lineage B.1.1.7, suggesting a common source of infection. These cases might represent a serious emergency because outbreaks can compromise frail patients with important concomitant diseases.

## Significant statement

The risk of infection occurs even for vaccinated healthcare workers. Cases occurring in healthcare workers can represent a serious emergency because outbreaks can compromise frail patients and elder population. Considering the recently identified new variants, it is essential to genotype the cases of infection outbreaks. These findings have implications for public health strategies aiming to increase the level of community control and protection.

## Introduction

The SARS-CoV-2 appeared first in Wuhan (China; Dec 2019) and quickly spread worldwide convincing the WHO to declare a COVID-19 pandemic (March 2020) [1, 2]. COVID-19 pandemic has caused 176.945.596 cases with 3.836.828 deaths and particularly 3.4 million cases and 127,153 deaths in Italy (June 2021; ourworldindata.org). After the European Medicines Agency (EMA) recommendations (Dec 21, 2020), the European Commission authorized the first vaccine against COVID-19 based on BNT162b2 mRNA (Comirnaty: Pfizer and BioNTech) [3]. Quickly, the Italian Medicines Agency (AIFA) authorized a national immunization campaign (Dec 22, 2020) starting with healthcare workers, excluding those healed from SARS-CoV-2 disease [3]. Clinical trials for BNT162b2 vaccine estimated an early immunization efficacy of 52.4% at first dose and 90.5% at 7 days after two doses in preventing COVID-19 [4]. A recent study estimated a 92% BNT162b2 effectiveness at 7 days after second dose (94% symptomatic COVID-19, 87% hospitalization, and 92% severe COVID-19) [5]. Despite this high efficacy and effectiveness of vaccination campaign, cases of SARS-CoV-2 infection still occur in vaccinated population [5].

Herein, we describe an outbreak of infection in healthcare workers receiving two doses of BNT162b2 vaccine.

## Methods

This study was carried out at the Pineta Grande Hospital (Castel, Volturno, Caserta, Italy). The intramural vaccination campaign started on Jan 30 (2021) and included the first dose of BNT162b2 mRNA vaccine followed by a second one after 21 days (Comirnaty, BioNTech-Pfizer, Mainz, Germany/New York, United States). Healthcare workers (656; 353F/303 M) were asked to join the study by signing the informed consent (prot n. R.CE 30/21; Azienda Sanitaria Locale Brindisi Ethical Committee, Brindisi, Italy). The study was conducted in accordance with the International Council of guidelines for good clinical practice of harmonization and the regulations of the Italian State, following the principles of Declaration of Helsinki. All specimen collections, specific extractions, and analysis were carried out according to COVID-19 safety instructions. As routine, healthcare workers were subjected to i. analysis of total IgG raised against the S1-RBD antigen and ii. nasopharyngeal swab tests made every 30 days. Anti-SARS-CoV-2 IgG quantification was performed by using the SARS-CoV-2 total chemiluminescent assay kit (COV2T) operating on the Advia Centaur XP System platform (Siemens Healthineers, Milan, Italy). Swabs were processed in an automated extraction platform (MagCore®, RBC Bioscience Corp., New Taipei City, Taiwan) and total RNA was quantified using Qubit Fluorometer (Life Technologies, Carlsbad, CA, USA). Real time-qPCR was carried out using the AMPLI-SARS-CoV-2 commercial kits (Dia-Chem srl, Napoli, Italy) targeting the virus nucleocapsid N1/N2 genes and the internal control RP gene, in a Real-Time PCR System (CFX 96; Bio-Rad, Hercules, CA, USA).

The genotyping was first performed by using AMPLI SARS-CoV-2 Variants commercial kit efficient for identifying 23 variants of Spike protein (Dia-Chem srl, Napoli, Italy). For variants’ confirmation, a direct sequencing analysis was performed. PCR primers were designed using Genomics Expression DNA Sequence Analysis Software referring to CoV-2 Spike glycoprotein (GenBank: NC_045512.2). Amplicons were purified (PCR Purification Kit; EuroClone ExoStar PCR-plate) and directly sequenced on both strands using Big Dye Terminator V3.1 Cycle Sequencing kit on an ABI3500 Genetic Analyzer (Applied Biosystems, Foster City, USA).

## Results

After vaccination, all healthcare workers were periodically monitored for specific virus immunoglobulins titration and subjected to molecular test for SARS-CoV-2 RNA expression every 30 days. This protocol was used for the purpose of identify asymptomatic or mild symptomatic SARS-CoV-2 positive subjects and assure a huge protection for healthcare personnel and patients.

Despite vaccination, five physicians were found positive to SARS-CoV-2 virus after 12 weeks from second dose, as detected by Real-Time PCR performed on RNA extracted from nasopharyngeal swab. The characteristics of these 5 healthcare workers are summarized in Table 1. Clinical assessment defined mild symptomatic SARS-CoV-2 positive subjects, according to a recommended schedule. Symptoms ranged from diarrhea (case 1), conjunctivitis (cases 2 and 5), muscle pain (cases 2, 4, and 5), breathlessness, fever higher than 38 (case 5) and lack of smells/tastes (case 3). All subjects had no concomitant diseases except for pre-obesity (cases 1, 2, and 3) and obesity (cases 4 and 5) (Table 1).

**Table 1:** Laboratory, vaccination, and clinical data of healthcare workers found positive for SARS-CoV-2

Case	1	2	3	4	5
*Demographic data*					
Age	48	22	25	63	56
Sex	M	F	F	M	F
BMI	28.08	28.68	28.59	33.59	35.15
*BNT162b2 mRNA vaccine*^*a*^					
dose I	*01/04/2021*	*01/07/2021*	*01/04/2021*	*01/07/2021*	*01/07/2021*
dose II	*01/25/2021*	*02/04/2021*	*02/01/2021*	*01/28/2021*	*01/28/2021*
*Antigenic test (AU/mL)*					
IgG, 15 days after dose I	239.7	726	155.6	407	726.5
IgG, 20 days after dose II	105	197.7	83.6	112	197.7
*Real-time PCR (Cts)*					
At the time of infection 3 months after dose I					
N1	22.0	24.6	15.7	26.2	23.6
N2	20.0	23.7	16.9	27.3	24.4
*Clinical symptoms*					
Fever	−	−	−	−	+
Cold	−	−	+	−	−
Conjunctivitis	−	+	−	−	+
Mild cough	−	−	−	+	+
Asthenia	+	−	−	−	−
Diarrhea	+	−	−	−	−
Headache	+	+	−	−	−
Muscle pain	−	+	−	+	+
Lack of smell/taste	−	−	+	−	−
Dizziness and breathlessness	−	−	+	−	−

*M*, male; *F*, female; *BMI* Body Max Index; *Cts* cycle threshold

^a^On January 2021, the hospital began the immunization campaign for 656 healthcare workers using the BNT162b2 mRNA vaccine (Comirnaty, BioNTech-Pfizer). Healthcare workers received the 2nd dose after 21 or 28 days and the five subjects resulted COVID-19 positive after 3 months after vaccination

**Fig. 1:**
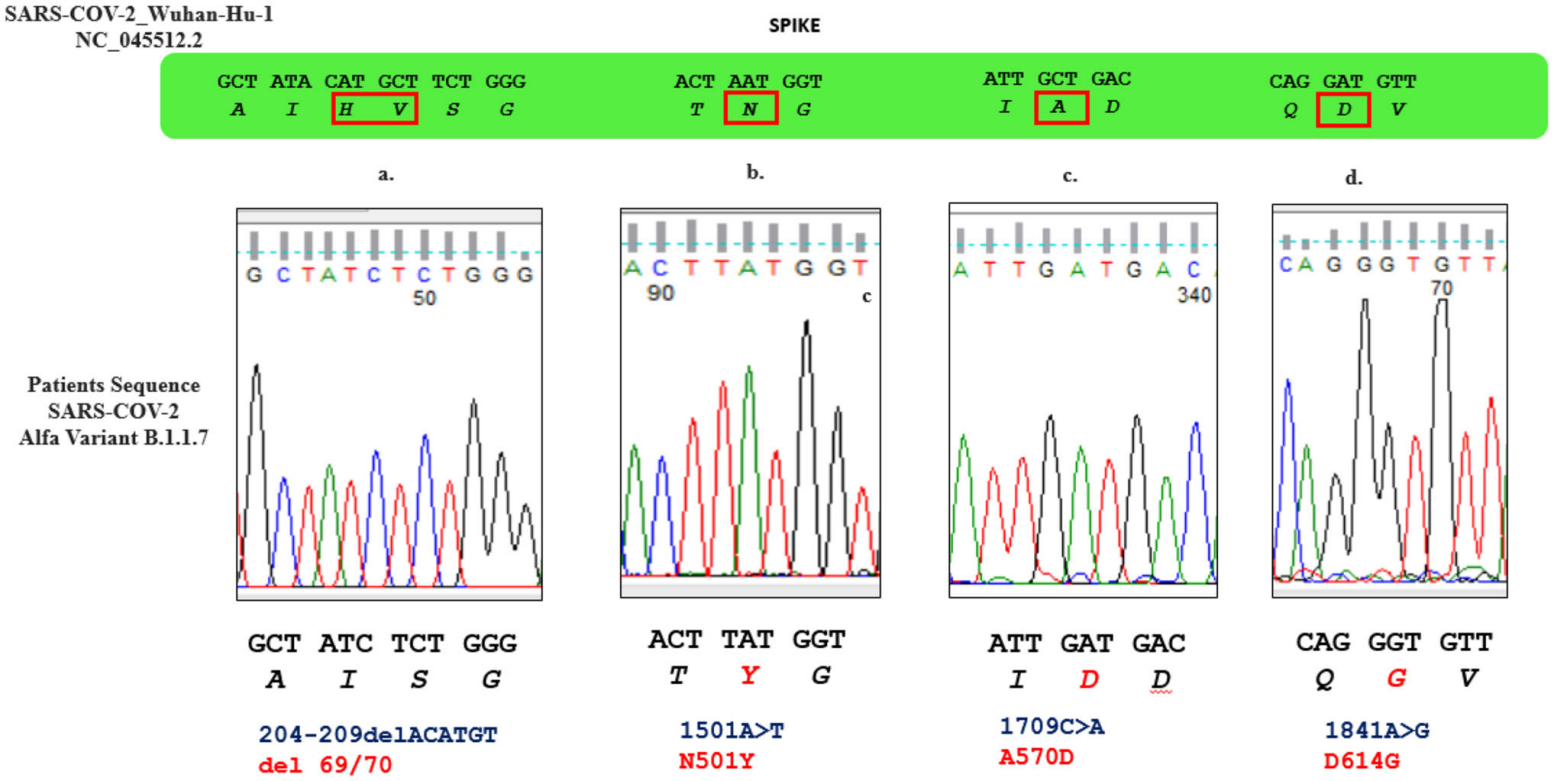
Identification of SARS-CoV-2 Spike-RBD mutations using Sanger method. Sections from the electropherograms showing the 69/70, N501Y, A570D, and D614G (a., b., c., d.) associated with SARS-CoV-2 Alfa Variant B.1.1.7

The genotyping performed first by Real-time PCR and then confirmed by direct sequencing proved the presence of del69/70, N501Y, A570D, and 1841A > G (D614G) variants, indicative of VOC 202,012/01-lineage B.1.1.7 [6], in all samples suggesting a common source of infection (Fig. 1).

Despite an epidemiologic investigation conducted by the hospital, neither the source nor modality of SARS-CoV-2 infection could be identified.

## Discussion

The development of COVID-19 disease was significantly reduced by worldwide vaccination campaign, although several recent studies highlighted the presence of outbreaks of infection among fully vaccinated healthcare workers. Amit and coworkers (Jan 2021) reported a positivity rate of 0.77% at 1–14 days and of 0.36% at 15–28 days after the first dose in Israeli healthcare workers immunized with BNT162b2 COVID-19 vaccine [6]*.* Merely, 22 out of 4,081 vaccinated healthcare workers (0.54%; Sheba Medical Center, Israel) developed COVID-19 between 1–10 days after immunization [7]. Further, data from Tel Aviv Sourasky Medical Center indicated a 1.18% and 0.50% frequency of infection in healthcare workers after first and second doses of BNT162b2 vaccine [7]. Keehner and coworkers (2021) reported about 1% total risk of testing positive for SARS-CoV-2 for vaccinated healthcare workers from universities UCSD (San Diego, USA) and UCLA (Los Angeles, USA) [8]. This higher incidence, as compared to previous observations [3, 4], was explained with the increased routine diagnostic testing and the greater possibility of encountering sources of infection in sanitary centers [8]. However, it is interesting to note that the frequency of positives decreased over time after vaccination: from 2.5% detected at 1–7 days after the first dose to 0.16% monitored at 15 days or more after second dose [8]. Further, an increased protection in vaccinated healthcare workers was observed in data collected from St Jude Children's Research Hospital (Memphis, Tennessee), showing a frequency of 1.34% after first dose and 0.36% after second dose, suggesting that the frequencies of infected healthcare workers can differ according to vaccination status [9]. The case series of 23,234 at the University of Texas Southwestern Medical Center (UTSW) and 22,729 healthcare workers in Northern California (Stanford University) showed similar frequencies [10]. The latest study reported the presence of the B.1.427/B.1.429 variant in 36% of cases [10]. Similarly, a study conducted in the Northern Italy (Brescia, April 2021) reported a 0.57% frequency of infection for 6904 vaccinated healthcare workers, with a lower risk (2.6-folds) than unvaccinated colleagues but still high (6.2 folds) as compared to common population [11].

Our finding performed on swabs from 5 healthcare workers tested positive for SARS-CoV-2 highlight the B.1.1.7 variant in four cases and the B1.525 variant in one case [11]. An outbreak of infection with VOC 202,012/01-lineage B.1.1.7 was previously described in two Italian physicians, one month after second dose (Southern Italy) [12].

Few studies have compared the efficacy of BNT162b2 vaccine in a population of healthcare workers including a control arm with unvaccinated subjects. To date, the SARS-CoV-2 Immunity and Reinfection Evaluation (SIREN) is the largest study from 104 UK hospitals comparing 20,641 vaccinated and 2683 unvaccinated healthcare workers [13]. During the 2-month follow-up period, 977 new infections were recorded in the unvaccinated cohort, while in the vaccinated group 71 and 9 new infections 21 days and 7 days after their first dose and second dose respectively were observed [13]. These results do not differ greatly from those reported in a study of 6493 (1090 unvaccinated and 5333 vaccinated) healthcare workers from Treviso (Italy) [14]. Although conducted with some differences in surveillance procedures and case identification, these two latter studies show good efficacy in preventing SARS-CoV-2 infection of BNT162b2 vaccine on healthcare workers with a 70–84% after 21 days from first dose and a 85–95% after 7 days from second dose [13, 14].

From our data, it is possible to state that vaccination has substantially reduced although not eliminated the risk of SARS-CoV-2 infection in healthcare workers. Modalities and susceptibilities to infection of vaccinated subjects are at present unknown, although prolonged exposure of physicians in a COVID ward during the third wave is a major predisposing factor. The observation of high viral load value in these subjects would suggest a source of infection with high viremia, as previously described [12]. It would be considering the greater contagiousness of VOC 202,012/01-lineage B.1.1.7 variant that was first identified in Italy (Dec 2020, [15]) and widely spread throughout the territory until reaching a prevalence of 88.1% in May 2021 [11, 12]. The different susceptibility to infection determined by genetic predisposition factors should not be overlooked [16–18].

Cases of healthcare workers infection can potentially have serious effects for transmission in working environments, including other staff members, frail or chronically ill patients, with implications not only at nosocomial level but also for entire healthcare system. Our data underline the importance of maintaining a high level of active and passive vigilance, continuing to use the specific personal protective equipment, correct distancing, and continuous laboratory screening programs.

Overall, these findings would suggest the necessity of a continuous SARS-CoV-2 surveillance even in the presence of full vaccination, to guarantee safety and healthiness of workers and patients [19].

## Data Availability

Raw data provided by each laboratory will be made available at reasonable request.

## Code availability

All software used for this study is mentioned in the text.
